# Impact of Second Opinions in Breast Cancer Diagnostics and Treatment: A Retrospective Analysis

**DOI:** 10.1245/s10434-019-07907-6

**Published:** 2019-10-11

**Authors:** E. Heeg, Y. A. Civil, M. A. Hillen, C. H. Smorenburg, L. A. E. Woerdeman, E. J. Groen, H. A. O. Winter-Warnars, M. T. F. D. Vrancken Peeters

**Affiliations:** 1 Department of Surgery, Leiden University Medical Centre, Leiden, The Netherlands; 2 Department of Surgery, The Netherlands Cancer Institute - Antoni van Leeuwenhoek Hospital, Amsterdam, The Netherlands; 3 Department of Medical Psychology, Amsterdam School of Public Health, Amsterdam University Medical Centers, Amsterdam, The Netherlands; 4 Department of Medical Oncology, The Netherlands Cancer Institute - Antoni van Leeuwenhoek Hospital, Amsterdam, The Netherlands; 5 Department of Plastic Surgery, The Netherlands Cancer Institute - Antoni van Leeuwenhoek Hospital, Amsterdam, The Netherlands; 6 Department of Pathology, The Netherlands Cancer Institute - Antoni van Leeuwenhoek Hospital, Amsterdam, The Netherlands; 7 Department of Radiology, The Netherlands Cancer Institute - Antoni van Leeuwenhoek Hospital, Amsterdam, The Netherlands

## Abstract

**Background:**

Breast cancer care is becoming increasingly complex, and patients with breast cancer are increasingly aware of the different treatment options, resulting in requests for second opinions (SOs). The current study investigates the impact of breast cancer SOs on final diagnosis and treatment in the Netherlands Cancer Institute (NCI) using a newly designed Breast Cancer Second Opinion (BCSO) classification system.

**Methods:**

Patients who visited the NCI for an SO between October 2015 and September 2016 were included. Demographics, diagnostics, and treatment proposals were compared between first and SO. Discrepancy was categorized using our BCSO classification system, categorizing SOs into (1) noncomparable, (2) identical, and (3) minor or (4) major discrepancy.

**Results:**

The majority of SOs (*n* = 591) were patient initiated (90.7%). A total of 121 patients underwent treatment prior to their SO, leaving 470 patients for assessment of discrepancies according to our BCSO classification system. More than 45% of these SOs resulted in at least one discrepancy, with comparable rates for physician- and patient-initiated SOs (42.5% vs. 45.6%, *p* = 0.708). Significantly more discrepancies were observed in patients with additional imaging (51.3% vs. 37.2%, *p* = 0.002) and biopsies (53.7% vs. 40.3%, *p* = 0.005). Almost 60% of all discrepancies were categorized as major (neoadjuvant systemic treatment instead of primary surgery, breast-conserving surgery instead of mastectomy, and proposing postmastectomy immediate breast reconstruction).

**Conclusions:**

Our findings show substantial differences in diagnostic and treatment options in breast cancer patients visiting the Netherlands Cancer Institute for an SO, thereby emphasizing more consensus for the indications of these treatment modalities.

Medical care for breast cancer patients is becoming increasingly complex, due to the emergence of patient- and tumor-tailored treatment and the wide variety of available treatment modalities. To address this complexity, all Dutch hospitals that provide breast cancer care have a multidisciplinary tumor board (MDT) where newly diagnosed breast cancer patients are discussed.^6^ Case review at a MDT is associated with improved breast cancer care.^1^^–^5 Additionally, these boards review second opinions (SOs) coming from different hospitals.

SOs can be initiated by patients themselves or by their physicians. Patient-initiated second opinions (PtSO) may be requested for a variety of reasons, but most frequently to achieve more certainty or reassurance about the diagnosis and/or treatment options provided by the first-opinion physician.^9^,^10^ Physician-initiated second opinions (PhSO) may occur either when the first-opinion physician seeks consultation or lacks expertise in complex care or when a patient is believed to have a psychological need for an additional opinion on their diagnosis and treatment plan.^11^,^12^

Of all patients diagnosed with some form of cancer, SOs are most frequently requested by patients with breast cancer.^7^,^8^ The rate of SOs among breast cancer patients reported in previous studies ranges between 9 and 20%.^10^,^13^^–^15 The demand for SOs may be increasing due to the increasing complexity of breast cancer treatment and subsequent complex decisions that patients and their physicians have to make. Furthermore, expansion of information provided by patient coalitions and media has enhanced patients’ awareness of available treatment options and the importance of shared decision-making.^16^

Besides their potential benefits, SOs have been suggested to have disadvantages: they may result in additional imaging and biopsies/biopsy procedures and could delay treatment onset.^7^,^17^,^18^ It has been debated whether an SO decreases patients’ uncertainty about the diagnosis and treatment plan.^15^ For physicians, SOs may increase workload and costs, and could result in a decrease of patients’ trust in them.^10^

A study by Mellink et al. of 317 Dutch patients diagnosed with various types of cancer reported that almost one-third of all SOs resulted in discrepancy regarding pathology and imaging interpretation or treatment advice between first and SO.^7^ In breast cancer patients, literature shows clinically relevant discrepancy in imaging interpretation of 13–38%^19^^–^22 and in pathology interpretation of 8–16%.^23^^–^25 Whether breast cancer SOs have a meaningful impact on clinical care is unknown. Previous studies focusing on breast cancer SOs used small sample sizes and heterogeneous definitions or no definition of what “clinical impact” of an SO entails.

In this study, we report on SOs for breast cancer in the Netherlands Cancer Institute-Antoni van Leeuwenhoek (NCI-AVL) hospital and their impact on final diagnosis and treatment using a newly designed Breast Cancer Second Opinion classification system. We analyze both PtSO and PhSO and describe (1) differences in interpretation of imaging and pathology and (2) the discrepancy in treatment proposals between first and SOs. Additionally, we analyze the impact of additional diagnostic procedures on these discrepancies.

## Methods

### Patient Population

All patients with breast cancer who visited the outpatient clinic of the Department of Surgical Oncology of the NCI-AVL for an SO between October 2015 and September 2016 were retrospectively selected. The NCI-AVL is a tertiary hospital with high expertise in cancer research and treatment. Patients were identified using a specific code in their medical file assigned when an SO request was mentioned in the referral letter. All patients who visit this outpatient clinic either have a clinical suspicion or have been diagnosed with breast cancer. Exclusion criteria for the current study were: the absence of a referral letter, referral by a general practitioner, and absence of a definitive diagnosis at first opinion or SO. Patients with a benign diagnosis at both first and second opinion were also excluded. Patients were categorized into two groups according to their “care phase” at SO to limit potential differences in discrepancy caused by received treatment: (1) patients who did not receive treatment prior to SO and (2) patients who had been treated at the time of the SO.

### Variables Studied

Information from the first opinion regarding the patients’ demographic characteristics, imaging and histopathologic results, and proposed treatment plan were retrieved from the referral letter, as well as from the imaging and pathology reports sent by the first opinion’s hospital. Whether the SO was patient or physician initiated was derived from the referral letter. We revised the pathology and imaging reports of the first opinion and included information obtained by additional imaging and pathology performed during the SO [e.g., magnetic resonance breast imaging (MRI), computed tomography (CT), or positron emission tomography (PET)-CT]. Additional diagnostic procedures that were strictly part of a trial protocol were not included. Genetic counseling was defined as “rapid genetic counseling and testing” (RGCT) when performed between diagnosis and primary surgery. Furthermore, we compared the treatment proposal at first opinion with the treatment undergone by the patient at the NCI-AVL, at the hospital providing the first opinion (after back-referral), or at a third hospital (in case of further referral after the SO). The treatment plan could be either (neo)adjuvant systemic treatment (NST) or primary surgery. Type of surgery was recorded for both the axilla [sentinel node (SN) biopsy, MARI procedure (marking of an axillary lymph node with a radioactive iodine seed before start of NST and removing this marked lymph node after NST), axillary lymph node dissection (ALND)] and the breast [breast-conserving surgery (BCS), mastectomy with or without immediate breast reconstruction (IBR)].

### Development of the Breast Cancer Second Opinion (BCSO) Classification to Assess Discrepancies

We composed a breast cancer SO (BCSO) classification system to quantify the degree of discrepancy between the first opinion and SO. This classification system was inspired by the surgical oncology SO classification suitable for patients diagnosed with any malignant neoplasm developed by Mellink et al.^7^ The BCSO classification system consists of four outcome categories: (1) incomparable, (2) identical, and (3) minor or (4) major discrepancy. The definitions of these categories were developed during five feedback sessions with a MDT. The categorization is based on discrepancy between first and SO in diagnostic findings, genetic screening, or treatment proposal. Patients were categorized as “minor discrepancy” when differences between the first and SO most likely had little impact on the treatment plan and prognosis. Patients were categorized as “major discrepancy” when differences most likely had a clinically relevant impact on patients’ treatment plan and prognosis. In case with both minor and major discrepancies, patients were categorized as having “major discrepancy.” Patients were categorized as incomparable when all clinicopathological findings, genetic screening, and the treatment proposal were unknown and when a patient received more than one treatment option. Patients were categorized as identical when the treatment proposal resulting from the second opinion was part of a trial (phase I/II) or when the treatment was a palliative option. A detailed description of the classification system is presented in Table 1.

**Table 1:** Breast cancer second opinion classification for discrepancy after a second opinion

Categorization	Description
Incomparable	Unknown or more than one treatment option given in first or second opinion Clinicopathological findings cannot be compared due to unknown findings in first or second opinion
Identical	Identical opinion on clinicopathological findings and treatment proposal Treatment proposal given by second opinion is part of a trial (phase I/II) Palliative treatment only option
Minor discrepancy	Minor change in findings from diagnostics, e.g. Clinical tumor stage (0 ↔ 1 or 1 ↔ 2 or 2 ↔ 3) Histological tumor type (ductal ↔ lobular) Differentiation grade (I ↔ II or II ↔ III) Genetic screening on urgent request instead of regular genetic screening Other changes not included in “major discrepancy”
Major discrepancy	Major change in diagnostics, e.g.: Benign instead of (pre)malignant Receptor status Differentiation grade (I ↔ III) Axillary lymph node involvement (N0 ↔ N+) Tumor stage (1 ↔ 3-4 or 2 ↔ 4 or 3 ↔ 4) Neoadjuvant treatment instead of primary surgery Change in type of surgery, e.g.: Breast-conserving instead of ablative surgery Postmastectomy immediate reconstruction instead of mastectomy only Change in treatment modality, e.g.: Adjuvant local treatment Adjuvant systemic treatment Systemic therapy instead of surgery

### Statistical Analysis

Descriptive statistics of patient and tumor characteristics and diagnostics were stratified according to the previously described two patient groups (i.e., untreated and treated prior to SO). All tests were two-sided, and *p* value < 0.05 was considered statistically significant. The discrepancy between first opinion and SO is described for patients according to the BSO classification system. The number of patients included per analysis might differ, as not all information of all patients was known at first and SO. All analyses were performed using SPSS^®^ version 24 (IBM, Armonk, NY, USA).

## Results

### Study Population

**Fig. 1:**
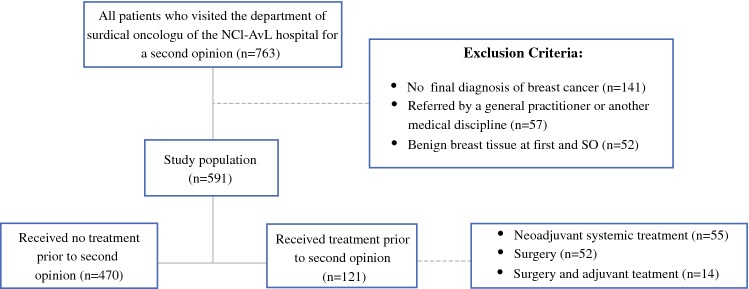
Number of patients with a second opinion for breast cancer at the Department of Surgical Oncology of the Netherlands Cancer Institute/Antoni van Leeuwenhoek hospital between October 2015 and September 2016. *Note*: some patients were excluded based on multiple exclusion criteria

We identified 763 patients who visited the breast cancer outpatient clinic of the NCI-AVL for a SO, of whom 591 patients met our eligibility criteria. Figure 1 shows the flowchart of patient selection and the treatment provided for those who received treatment at first opinion.

Table 2 presents patient and tumor characteristics as described in the referral letter. The vast majority of SOs were patient initiated (90.7%). The mean age of all patients was 50.9 (range 29–82) years, and two patients were male. Of all patients diagnosed with invasive breast cancer, 51.5% were diagnosed with hormone receptor (HR)-positive (estrogen or progesterone) and human epidermal growth factor receptor 2 (HER2)-negative tumors, 15.0% with HR-negative and HER2-positive tumors, 14.2% with triple-negative tumors, and 19.3% with an unknown subtype. Of all patients, 61.9% were classified as stage I or II. Only 3.7% had metastatic disease.

**Table 2:** Patient and tumor characteristics described in the referral letter at first opinion (*n* = 591)

			Treatment prior to second opinion	
		All patients (*n* = 591)	No (*n* = 470)	Yes (*n* = 121)
Second opinion initiated by	Physician	55 (9.3%)	40 (8.5%)	15 (12.4%)
	Patient	536 (90.7%)	430 (91.5%)	106 (87.6%)
Age at diagnosis (years)	Mean (SD)	51 (12)	51 (12)	51 (11)
Gender	Female	589 (99.7%)	469 (99.8%)	120 (99.2%)
ASA classification	I	475 (80.4%)	376 (80%)	99 (81.8%)
	II	92 (15.6%)	79 (16.8%)	13 (10.7%)
	III	24 (4.1%)	15 (3.2%)	9 (7.4%)
Diagnosis	DCIS	75 (12.7%)	66 (14.0%)	9 (7.4%)
	Invasive ± DCIS	507 (85.8%)	399 (84.9%)	108 (89.3%)
	Other	9 (1.5%)	5 (1.1%)	4 (3.3%)
Histological tumor type	Ductal	443 (75%)	356 (75.7%)	87 (71.9%)
	Lobular	88 (14.9%)	62 (13.2%)	26 (21.5%)
	Combination of ductal/lobular	47 (8%)	39 (8.3%)	8 (6.6%)
	Unknown	13 (2.2%)	13 (2.8%)	0 (0.0%)
Differentiation grade	I	64 (10.8%)	51 (10.9%)	13 (10.7%)
	II	178 (30.1%)	121 (25.7%)	57 (47.1%)
	III	98 (16.6%)	72 (15.3%)	26 (21.5%)
	Unknown	251 (42.5%)	226 (48.1%)	25 (20.7%)
Receptor status^a^	Triple-negative	72 (14.2%)	58 (14.5%)	21 (13.0%)
	HER2+	76 (15.0%)	55 (13.8%)	16 (19.4%)
	HR+ (ER and/or PR) and HER2–	261 (51.5%)	195 (48.9%)	66 (61.1%)
	Unknown	98 (19.3%)	91 (22.8%)	7 (6.5%)
Stage	0	76 (12.9%)	70 (14.9%)	6 (5.0%)
	I	153 (25.9%)	143 (30.4%)	10 (8.3%)
	II	213 (36.0%)	178 (37.9%)	35 (28.9%)
	III	76 (12.9%)	49 (10.4%)	27 (22.3%)
	IV	22 (3.7%)	15 (3.2%)	7 (5.8%)
	Unknown	51 (8.6%)	15 (3.2%)	36 (29.8%)
Trial participation	Yes	131 (22.2%)	119 (25.3%)	12 (9.9%)

*SD* standard deviation, *ASA* American Society of Anesthesiologists, *DCIS* ductal carcinoma in situ, *HR* hormone receptors, *HER2* human epidermal growth factor receptor 2

^a^Invasive breast cancer

Of the 470 patients, untreated prior to SO, 269 (57.2%) underwent MRI, 187 (39.8%) underwent CT/PET-CT, and 175 (37.2%) patients underwent at least one additional biopsy at the NCI-AVL. Of the 121 patients who had already started their treatment at first opinion, 25 (20.7%) underwent MRI, 22 (18.2%) underwent CT/PET-CT, and 14 (11.6%) underwent additional biopsies at the NCI-AVL. Of the 470 patients, 119 (25.3%) participated in a trial at SO, of whom 17 participated in a phase 1/2 trial.

### Discrepancy According to the Breast Cancer Second Opinion Classification

Discrepancy between the first and SO according to the BCSO classification could be assessed for patients who had not received treatment prior to SO, as previous treatment would be expected to give rise to changes in diagnostic findings and further treatment proposal.

In 213 (45.3%) of the 470 patients untreated prior to SO, a discrepancy between first opinion and SO was observed. Of these discrepancies, 76 (16.2%) were minor and 137 (29.1%) were major. Of the 137 patients with a major discrepancy, 38 (27.7%) patients additionally had a minor discrepancy. In total, 257 (54.7%) patients were classified as identical.

SOs initiated by a physician did not result in higher discrepancy rates compared with those initiated by a patient (42.5% vs. 45.6%, respectively, *p* = 0.708). Participation in trials, besides phase 1/2, did not result in more discrepancy, as discrepancies were seen in 52.9% patients enrolled in a trial versus 42.7%% in patients who did not participate in a trial (*p* = 0.053). Table 3 presents the discrepancy rates based on our BCSO classification.

**Table 3:** Discrepancy between first and second opinion according to breast cancer second opinion classification of 470 patients who had not yet received treatment prior to second opinion

	Discrepancy		
	No	Yes	Incomparable
Minor discrepancies^a^	394 (83.8%)	76 (16.2%)	
Stage (e.g., I ⇔ II)	385 (84.6%)	70 (15.4%)	15
Histological tumor type (e.g., ductal ⇔ lobular)	428 (94.7%)	24 (5.3%)	18
Differentiation grade (e.g., I ⇔ II)	214 (88.1%)	29 (11.9%)	227
Genetic screening (e.g., genetic screening after surgery ⇔ RGCT)	19 (79.2%)	5 (20.8%)	446
Major discrepancies^a^	333 (70.9%)	137 (29.1%)	
Malignancy (e.g., benign ⇔ malignant)	468 (99.6%)	2 (0.4%)	0
Receptor status^b^ (e.g., triple negative ⇔ HER2 positive)	301 (98.7%)	4 (1.3%)	94
Differentiation grade (e.g., I ⇔ III)	242 (99.6%)	1 (0.4%)	227
Lymph node involvement (e.g., N0 ⇔ N1)	430 (97.5%)	11 (2.5%)	29
Stage (e.g., I ⇔ III)	443 (97.4%)	12 (2.6%)	15
Neoadjuvant therapy (e.g., neoadjuvant therapy ⇔ primary surgery)	258 (77.2%)	76 (22.8%)	136
First surgery (e.g., mastectomy ⇔ breast-conserving surgery)	266 (89.9%)	30 (10.1%)	174
Reconstruction^c^ (e.g., mastectomy only ⇔ mastectomy with IBR)	50 (61.7%)	31 (38.3%)	58

Not comparable may be due to missing information in first or second opinion

*RGCT* rapid genetic counseling and testing, *IBR* immediate breast reconstruction

^a^In case of both minor and major discrepancies, patients were categorized as having a “major discrepancy.” Total or subgroup total discrepancy can differ with different categories added up as patients are counted once

^b^Invasive breast cancer

^c^Patients who underwent a mastectomy as their first surgical therapy

Minor discrepancies between first opinion and SO were mostly found in tumor staging, which occurred in 70 (15.4%) patients. Major discrepancies mostly concerned differences in primary treatment proposal. A total of 76 (22.8%) out of 334 patients with a primary treatment proposed at first opinion received a different primary treatment compared with the proposed treatment at first opinion. A total of 59 (17.7%) patients received NST instead of primary surgery, and 17 (5.1%) patients underwent primary surgery instead of NST. Other common major discrepancies were observed in type of first surgery and in use of IBR in patients who underwent mastectomy. Information regarding the proposed treatment of the axilla (SN, MARI, or ALND) was not well documented in the referral letters and could therefore not be compared. Genetic counseling was not frequently mentioned in the referral letter and was only known in 24 patients at first and SO. Two (0.4%) patients had a change in their primary diagnosis from malignant at first opinion to benign at SO.

Of the patients who received additional biopsy at SO, discrepancy between first and SO was observed in histology in 10.2% (out of 167 patients), differentiation grade in 23.2% (out of 82 patients), invasiveness in 1.1% (out of 174 patients), tumor stage in 26.7% (out of 172 patients), and receptor status in 3.4% (out of 119 patients). Additional diagnostics at SO resulted in a significantly higher discrepancy rate: discrepancy (minor or major) was more common in patients who received additional imaging (51.3% vs. 37.2%, *p* = 0.002) or biopsy (53.7% vs. 40.3%, *p* = 0.005) at SO compared with those who did not.

### Location of Further Treatment after the Second Opinion

A total of 293 (62.3%) patients of the 470 patients untreated prior to SO remained at the NCI-AVL after the SO for all further treatment, while 92 (19.6%) patients remained for part of the treatment and 85 (18.1%) patients returned to the hospital of first opinion for the entire treatment. The majority (95.7%) of the patients who stayed in our hospital for a part of the treatment underwent surgery at the NCI-AVL and received systemic therapy at the hospital of first opinion. Patients with a discrepancy (minor or major) between first opinion and SO more often remained in the NCI-AVL for all further treatment as compared with patients without discrepancy (46.4% vs. 32.9%, *p* = 0.027).

Of the 121 patients who had received treatment at first opinion, 41 (33.9%) patients remained at the NCI-AVL after SO for all further treatment, 28 (23.1%) patients remained for part of the treatment, and 52 (43.0%) patients returned to the hospital of first opinion for the entire treatment.

## Discussion

In this retrospective series at a tertiary cancer center in The Netherlands, we observed that an SO for breast cancer in untreated patients resulted in at least one discrepancy in diagnostic findings or therapeutic advice in 45% of patients, 29% of which were major. The most common discrepancies were observed regarding primary treatment proposal (NST instead of primary surgery), type of surgery (BCS instead of mastectomy), and the use of IBR in those who underwent mastectomy (IBR instead of mastectomy only). The discrepancy rate (minor or major) was significantly higher in patients who received additional imaging and biopsies at SO compared with those who did not. Our newly designed BCSO classification can be used in a reproducible manner in future studies to assess the clinical impact of SOs, enabling comparison of results between studies.

It could be expected that improvements in care and increased standardization of breast cancer management in the last decade might have resulted in reduced discrepancy between first and SOs. However, the discrepancy rate in the current study is higher than the 16% minor and 16% major discrepancy rate for SOs reported in 2006 in a sample of Dutch cancer patients, of whom 72% had breast cancer.^7^ Our higher discrepancy rate might be explained by differences in the definition of discrepancy, as the classification system used in the former study was applicable to patients with several types of cancer and thus was different from our BCSO classification system.^7^

The highly variable definitions of discrepancy complicate comparison of the present results with other previous findings in breast cancer, in which discrepancy rates between 3 and 43% were reported.^1^,^4^,^5^,^7^,^19^,^23^,^26^ The BCSO classification for discrepancy developed in the current study will enable detailed and reproducible comparisons between first opinions and SOs in the future.

To the best of the authors’ knowledge, this is the first study to report discrepancy in proposed primary treatment between first and SO in detail, being NST or surgical therapy. We found that almost 23% of patients received a different primary treatment proposal (NST or primary surgery). Large variation exists between Dutch hospitals in use of NST, due to divergent expert opinions on the indications for NST.^27^,^28^ This disagreement between physicians on the indications for NST might partly explain the discrepancy in proposed primary treatment. These results emphasize that more consensus is needed to reduce the variation in the indications for NST.

The SOs evaluated in the current study were provided in a tertiary hospital with high expertise on breast cancer care.^6^ Previous studies have suggested that expertise in treatments such as the use of NST and IBR might be indications for an SO or a change of hospital after diagnosis.^9^,^29^ This is partly true, as results from a national audit show that the use of NST and IBR in the NCI-AVL is above the national Dutch average.^6^ On the other hand, the number of patients who received NST followed by BCS was comparable between patients who remained in the NCI-AVL and those who returned to the hospital of first opinion. The discrepancy in use of IBR in the current study might partly be explained by the ongoing discussion on the timing of breast reconstruction when adjuvant radiation therapy is indicated.^30^,^31^ Other hospitals may have a different policy on performing IBR when the patient needs adjuvant radiation.

The current finding that discrepancy rates were higher for patients who received additional imaging at SO emphasizes the value of SO review and importance of additional imaging. This is in concordance with previous studies focusing on the impact of additional imaging procedures.^1^,^19^,^20^ Nonetheless, it was not always clear in previous studies whether diagnostic discrepancies resulted in an alteration of treatment.

A discrepancy in genetic counseling has not been analyzed in previous studies on SOs before. Although genetic counseling could be compared in only 24 patients, we observed that 5 (20.8%) patients received RGCT instead of postoperative genetic screening. Deptite the fact that none of these latter patients had a discrepancy in the type of surgery, use of genetic counseling before surgery could influence decision-making regarding primary surgery and timing of risk-reducing contralateral mastectomy.^32^ Unfortunately, the small number of patients does not allow any hard conclusions to be drawn regarding the causality between SOs and genetic counseling.

This is the first study comparing the discrepancy rate between patient- and physician-initiated SOs. We found comparable rates of minor and major discrepancies between both types of SOs. A surprising finding in the current study is that, out of the 257 patients with an identical SO, only 22.2% returned to the hospital providing the first opinion for the entire treatment. These findings are not in line with findings by Mellink et al., who reported that 85% of patients with an identical SO returned to the first opinion.^7^ Although not the focus of our study, this could on one hand suggest that there is room for improvement at the first opinion to encourage patients in seeking an SO and reassuring them that they can return after the SO. On the other hand, it could indicate that the hospital providing the SO should stimulate back-referral of patients to the hospital of first opinion and aim to enhance patients’ trust in the physician who provided the first opinion.

The high discrepancy rates reported in our study should be interpreted with caution. Although the discrepancy rate might seem high, it may primarily show the complexity of breast cancer management: Not every discrepancy is associated with better care; some discrepancies might reflect interobserver variability and institutional preferences, and may not be based on guideline recommendations. The findings of the current study are subject to at least three limitations. First, it is important to bear in mind that an SO can be an opinion based on information on top of the information from the first opinion, as is the case with the use of additional imaging by an SO. It is unknown whether the first opinion would also have performed the additional imaging resulting in an altered diagnosis or treatment proposal. Nonetheless, almost 97% of included SOs received a diagnosis or treatment proposal from the first opinion based on the information available at that moment. Second, our retrospective study design does not allow any hard conclusions to be drawn regarding the causality of discrepancy in imaging and altered treatment. Thirdly, our findings cannot be extrapolated to all breast cancer SOs, as the study was conducted in a tertiary setting. However, the developed BCSO classification allows better comparison of discrepancy in a reproducible manner.

## Conclusions

Our study showed, with the use of the newly developed BCSO classification, a substantial impact of SOs on breast cancer diagnostics and treatment. Major discrepancies mainly concerned primary treatment, type of first surgery, and use of IBR, thereby emphasizing the importance of more consensus for the indications of these treatment modalities. Future studies focusing on the impact of SOs could use the BCSO classification to assess discrepancy between first opinion and SO in a detailed and reproducible way. With the increasing use of nationwide cancer registries, future studies could include SOs from all different types of hospitals, which could validate the current single-center evidence on the impact of SOs.
